# Effect of isoniazid preventive therapy on tuberculosis or death in persons with HIV: a retrospective cohort study

**DOI:** 10.1186/s12879-015-1089-3

**Published:** 2015-08-13

**Authors:** Henok Tadesse Ayele, Maaike SM van Mourik, Marc JM Bonten

**Affiliations:** Julius Center for Health Sciences and Primary Care, Infectious Diseases Epidemiology, University Medical Center Utrecht, Utrecht, The Netherlands; Department of Medical Microbiology and Infection Control, University Medical Center Utrecht, Utrecht, The Netherlands; Department of Public Health, Dilla University College of Medicine & Health Sciences and Referral Hospital, Dilla, Gedeo Zone Ethiopia

## Abstract

**Background:**

Isoniazid preventive therapy (IPT) is a recommended strategy for prevention of tuberculosis (TB) in persons with Human Immunodeficiency Virus (HIV) although the benefits have not been unequivocally demonstrated in routine clinical practice with widespread ART adoption. Therefore, we assessed the effectiveness of IPT in prevention of TB or death in patients treated with antiretroviral therapy (ART) in a chronic care setting.

**Methods:**

Retrospective cohort study of HIV patients enrolled in chronic care from 2007 to 2013. Eligible participants were HIV infected subjects (age > 15 years) with no (history of) TB. The combined effect of IPT and ART on the composite outcome (TB or death) was estimated using time-dependent Cox regression with adjustment for baseline covariates.

**Results:**

1,922 patients were included, 374 (19.4 %) received IPT and 258 (13.4 %) developed TB or deceased. The median follow-up duration of the cohort was 839 days, with a total of 5491 person years. In unadjusted analysis, the combination of IPT and ART lowered the hazard of TB or death by 65 % [HR = 0.35; 95 % CI (0.16, 0.77)] compared to ART alone. Even after adjustment for confounders, the combined effect of ART and IPT resulted in a 60 % hazard reduction of TB or death in comparison to participants who received ART without IPT [HR = 0.40; 95 % CI (0.18, 0.87)]. The IPT-specific benefit in patients not receiving ART could not be reliably estimated due to high rates of ART adoption.

**Conclusion:**

The combined effect of IPT and ART to prevent TB or death in HIV patients in a non-experimental setting in comparison to ART alone was estimated to be 60 %.

## Background

The World Health Organization (WHO) has estimated that in 2013 nine million people developed tuberculosis (TB) with 1.5 million associated deaths, including 360.000 among persons with Human Immunodeficiency Virus [1]. Although the introduction of anti-TB treatment in the early 1950s reduced the disease burden in developed countries, TB remains a major global health problem in many parts of the world, especially in Sub-Saharan African (SSA) countries [2].

Only a minority of people exposed to *Mycobacterium tuberculosis (MTB)* develop active TB [3]. In healthy individuals, the immune system controls the infection and patients can remain asymptomatic for prolonged periods, a so-called latent infection, that may progress to active TB with waning immuntity [4]. The HIV epidemic has changed the dynamics of TB disease [5]. HIV infection increases the risk of progression to TB disease after primary exposure [6], reinfection [7, 8] or from latent TB [9, 10], and thereby the incidence and prevalence of actual TB [11, 12], and the risk of transmission to uninfected persons [13, 14].

In 2004, the WHO and Stop TB Partnership developed an interim policy on collaborative TB/HIV activities [15]. The four main TB prevention methods include intensified case finding, isoniazid preventive therapy (IPT), tuberculosis infection control, and antiretroviral therapy (ART) [15]. So far, only ART has been implemented on a large scale [16]. To improve TB prevention and treatment, WHO launched an intervention package which is comprised of IPT, intensified case finding, and infection control (three I’s) in parallel with ART. [17] IPT has proven preventive efficacy in both non-HIV-infected and HIV-infected individuals [18], however, only 32.5 % of eligible individuals received IPT worldwide in 2012 [16, 19]. In a meta-analysis of eight placebo-controlled trials involving 4136 HIV-infected participants [20], the overall efficacy of IPT in preventing TB was 33 % although reductions among Tuberculin Skin Test (TST) negative patients were not statistically significant [20]. In a recently published study parallel therapy with IPT and ART was more effective than the individual therapies alone [21].

Importantly, most of the empirical evidence on efficacy of IPT is derived from clinical trials conducted in controlled settings. Evidence is lacking from routine health care settings and some of the observational studies conducted are limited by methodological concerns; for example, not all incorporate the time varying nature of ART and IPT exposure. In addition, some studies do not account for death as a competing event when estimating the effect of IPT on TB incidence. Hence, this retrospective cohort study aims to assess the effectiveness of IPT in patients receiving ART in a routine care setting.

## Methods

### Setting

This retrospective cohort study was conducted at Dilla University Referral Hospital in Ethiopia. Ethiopia has adopted ART in 2005 [22] and implemented the combined TB/HIV program including IPT in 2007 [23].

### Study design & study population

**Fig 1:**
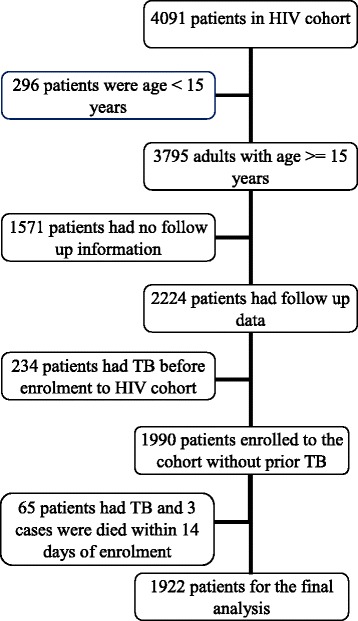
The schematic presentation of subjects selection for the final analysis from HIV cohort of Dilla University Referral Hospital

This study included all patients who had been treated in the TB and HIV/AIDS continuum of care between January 2007 and August 2013. Patients aged less than 15 years, who did not have follow up information after first entry to the cohort, had TB before enrolment to the chronic care unit, or either died or were diagnosed with TB within 14 days of enrolment were excluded (Fig. 1).

Ethical approval was obtained from Dilla University College of Health and Medical Sciences ethical review board and Dilla University Hospital, Clinical and Practical training director office.

### Data collection

Information on patient characteristics, severity of disease, treatments and outcomes was retrieved from patient medical records by data clerks, with verification of accuracy in a random sample. Data were entered in the EpiData template version 3.0 (EpiData Association, Odense, Denmark) and were anonymized prior to analysis. Baseline covariates included gender, age, ever receiving cotrimoxazole prophylaxis, ART, weight, CD4 count, WHO stage of HIV infection, and functional status. Follow-up visits for IPT and ART were monthly or every two months, respectively. Further regular follow-up visits were planned every six months for the first year and once a year thereafter. During these visits CD4 counts, body weight, functional status and WHO stage were recorded.

### Outcome

The outcome of this study was the occurrence of TB or all-cause mortality during follow-up. According to the routine clinical guideline, TB was ascertained either by a positive sputum smear, suggestive chest X-ray, suggestive pathology or a positive sputum culture [23]. The date of death was determined by review of hospital charts or interview of relatives. In the absence of the above events, subjects were censored if they were lost to follow-up or if they were alive at the end of the study period (August 2013).

### Interventions (Exposure)

IPT and ART were the determinants of interest in this study and they were initiated according to local routine clinical practice. All HIV patients without TB or previous history of TB were eligible for a course of IPT (300 mg for six months), and it was provided free of charge. The eligibility for IPT was determined by broad clinical assessment to rule out TB up to 2010 [24]. Starting 2010, more stringent screening criteria were used to rule out pre-existing TB and IPT was started in patients without current cough, night sweats, weight loss, or fever, in accordance with WHO recommendations [25]. TST testing was not required as screening tool in both periods.

According to the national ART guidelines, patients were eligible for ART when they developed more advanced HIV disease (WHO stage III or higher) or had CD4 counts less than 200 cells/mm^3^ [3, 26].

### Statistical methods

Data integrity was assessed and descriptive statistics were generated. At baseline less than 5 % of data were missing for WHO HIV disease stage, WHO functional status, CD4 count and body weight; missing observations were imputed by multiple imputation and effect estimates were pooled using Rubin’s rule [27]. Cox regression was used to estimate unadjusted and adjusted hazard ratios for the composite outcome of TB or death. IPT and ART treatment status was included as time-varying exposure and the estimates were adjusted for the following baseline covariates: age, gender, cotrimoxazole prophylaxis, weight, CD4 count, WHO stage and functional status. If a patient on ART treatment began IPT, person-time no longer accumulated in the ART alone category, but rather person-time and subsequent events contributed in the category labeled ‘on ART and treated with IPT’. This person-time movement also occurred for the small number of patients treated with IPT who then began ART therapy. Patients who completed treatment with IPT remained in the IPT treatment category until the end of follow-up. Incidence rates of TB, death, and composite outcome were calculated in each exposure category. A considerable proportion of data were missing during follow up and adjusted analysis was not possible for covariates other than those measured at baseline. Treatment with ART but not IPT was considered the reference group. The assumptions of Cox regression model were assessed by examining Schoenfeld residuals for proportional hazards, dfbeta’s for influential observation, and martingale residuals for non-linearity [28]. The level of significance was taken at p ≤0.05. All analysis was conducted in SPSS version 20 [29] and R version 3.0.2 [30].

## Results

Of the 4,091 patients enrolled in the comprehensive follow-up program, 1,922 were included in the final analysis (Fig. 1). Patients were excluded for one of the following reasons: age less than 15 years (n = 296), lack of follow-up information (n = 1,571), occurrence of TB before enrolment to the chronic care unit (n = 234), TB or death within the first fourteen days of enrolment to the chronic care unit (n = 68). The were no statistically significant differences in mean age and gender of cases excluded due to a lack of follow-up information, those participants lost to follow up and the participants remaining in the cohort.

Three hundred seventy four (19.5 %) participants received IPT during their follow up in the care program. The majority of subjects (55.8 %) were females and close to two third of the study subjects (60.5 %) were younger than 34 years (Table 1). Eighty five percent of the study subjects (n = 1635) were not on ART at baseline, but almost all (n = 1919) received or started ART during follow-up. In addition to IPT prophylaxis, many patients (84.9 %) had received prophylactic co-trimoxazole. The majority of the participants (64.3 %) had stage III HIV disease at baseline. Most subjects were either at working (58 %) or at ambulatory (36.4 %) functional status according to the WHO operational definition. Sixty-five percent of the subjects had a CD4 count less than 199 cells/mm^3^ and less than 4 % of the participants had CD4 counts above 350 cells/mm^3^. Almost half of the participants (n = 879) weighed less than 50 kgs (Table 1).

**Table 1:** The Socio-demographic and baseline characteristics in IPT and non IPT cohorts in Dilla University Referral Hospital

Baseline covariates		Non IPT cohort	IPT cohort	Total
		N = 1548	N = 374	N = 1922
		No. (%)	No. (%)	No. (%)
Gender	Female	848 (54.8)	225 (11.768.2)	1073 (55.8)
Age group	15 – 25 years	333 (21.5)	78 (20.9)	411 (21.4)
	26 – 29 years	238 (15.4)	63 (16.8)	301 (15.7)
	30 – 34 years	355 (22.9)	94 (25.1)	449 (23.4)
	35 – 39 years	268 (17.3)	58 (15.5)	326 (17.0)
	40 – 99 years	354 (22.9)	81 (21.7)	435 (22.6)
ART at baseline	No	1307 (84.4)	328 (87.7)	1635 (85.1)
	Yes	241 (15.6)	46 (12.3)	287 (14.9)
Cotrimoxazole (ever use)*	Yes	1290 (83.3)	341 (91.2)	1631 (84.9)
WHO stage of HIV disease*	Stage I	141 (9.1)	39 (10.4)	180 (9.4)
	Stage II	247 (16.0)	78 (20.9)	325 (16.9)
	Stage III	1000 (64.6)	236 (63.1)	1236 (64.3)
	Stage IV	160 (10.3)	21 (5.6)	181 (9.4)
WHO functional status*	Ambulatory	590 (38.1)	110 (29.4)	700 (36.4)
	Bedridden	99 (6.4)	8 (2.1)	107 (5.6)
	Working	859 (55.5)	256 (68.4)	1115 (58.0)
CD4*	0 – 99 cells/mm^3^	477 (30.8)	76 (20.3)	553 (28.8)
	100 – 199 cells/mm^3^	561 (36.3)	142 (38.0)	703 (36.6)
	200 - 349 cells/mm^3^	446 (28.8)	145 (38.8)	591 (30.8)
	350 + cells/mm^3^	63 (4.1)	11 (2.9)	74 (3.9)
Body weight	< = 50 Kgs.	726 (47.5)	153 (41.6)	879 (46.4)
	50-74 Kgs.	763 (50.0)	204 (55.4)	967 (51.0)
	75 + Kgs.	38 (2.5)	11 (3.0)	49 (2.6)
Transfer in	yes	160 (10.3)	36 (9.6)	196 (10.2)
Point of entry to care (Entry from)	Referred from the same hospital	1197 (77.3)	296 (79.1)	1493 (77.7)
	Self-referred	29 (1.9)	6 (1.6)	35 (1.8)
	Referred from other health facilities	322 (20.8)	72 (19.3)	394 (20.5)
Outcomes	Censored	1301 (84.0)	363 (97.1)	1664 (86.6)
	TB	105 (6.8)	5 (1.3)	110 (5.7)
	Death	142 (9.2)	6 (1.6)	148 (7.7)
Composite outcome	TB or death	247 (16.0)	11 (2.9)	258 (13.4)

*:p value < 0.05. ***ART*** Antiretroviral therapy, ***HIV*** Human Immunodeficiency Virus, ***IPT*** Isoniazid Preventive Therapy, ***N*** Number of people, ***TB*** Tuberculosis, and ***WHO*** World Health Organization

Five hundred seventy eight participants were lost to follow-up during the study period and were censored, together with the 460 participants alive at the end of the study (Table 2). The median follow-up duration of the cohort was 839 days with 5491.2 person-years (PYs) in total. In total, 110 patients developed TB and 148 died during the course of the study. TB was diagnosed based on a positive sputum smear (n = 81), suggestive chest X-ray (n = 13), suggestive pathology (n = 12) or a positive sputum culture (n = 4). The incidence of TB (6.6 per 100 PYs), death (9.5 per 100 PYs), and the composite outcome (16.1 per 100 PYs) was higher within the first six months, with a decreasing trends in the following months (Table 2).

**Table 2:** Follow up information of HIV cohort in Dilla University Referral Hospital from January 2007 to August 2013

Follow up time	Total participants on follow up n (PYs)	IPT started	ART started	TB Diagnosed n (Per 100 PYs)	Death Reported n (per 100 PYs)	Composite outcome n (per 100 PYs)	Lost to follow up n (per 100 PYs)	Censored alive
Baseline (t = 0)	1922 (0)	12	289	0 (0)	0 (0)	0 (0)	0 (0)	0
6th month	1519 (865.2)	43	1293	57 (6.59)	82 (9.48)	139 (16.07)	175 (20.22)	89
12th month	1300 (703.1)	50	109	16 (2.28)	21 (2.99)	37 (5.26)	143 (20.34)	39
24th month	1028 (1165.7)	94	104	19 (1.63)	15 (1.29)	34 (2.92)	130 (11.15)	108
36th month	780 (893.7)	81	60	8 (0.90)	13 (1.46)	21 (2.35)	66 (7.39)	161
48th month and beyond	626 (1863.4)	94	64	10 (0.54)	17 (0.91)	27 (1.45)	64 (3.44)	63
Total	(5491.2)	374	1919	110 (2.00)	148 (2.70)	258 (4.70)	578 (10.53)	460

***ART*** Antiretroviral therapy, ***IPT*** Isoniazid Preventive Therapy, ***n*** Number of people, ***PYs*** Person years, and ***TB*** Tuberculosis

Among the 374 patients receiving IPT, 45 (12 %) did not complete six months of IPT: 34 were censored alive during their IPT treatment, 7 were lost to follow up and 4 had an outcome (TB or death). Overall, the rate of TB was higher in ART naïve participants (8.05 per 100 PYs) followed by ART naïve participants yet treated with IPT (5.20 per 100 PYs) compared to those treated with both ART and IPT (Table 3). In the crude analysis, male patients were at higher risk of developing TB or dying [HR = 1.44; 95 % CI (1.12, 1.83)] (Table 4). Furthermore, patients who were bed-ridden and those with more advanced HIV disease (WHO stage III or higher) at baseline had an increased hazard of developing TB or death in comparison to ambulatory patients or those with early disease stages, respectively. Increasing baseline patient body weight decreased the hazard of developing TB or death by 2 % [HR = 0.98; 95 % CI (0.97, 0.99)]. With respect to the patients’ entry point of care, those referred from other health facilities had a lower hazard to develop TB or die [HR = 0.61; 95 % CI (0.41, 0.87)] in comparison to patients referred from different departments of the same hospital.

**Table 3:** Incidence rate of endpoints (TB, death, and composite) in IPT and ART exposure category in HIV cohort of Dilla University Hospital from January 2007 to August 2013

Exposure status	Person years	TB n (per 100 PYs)	Death n (per 100 PYs)	Composite n (per 100 PYs)
On ART, no IPT	3922.6	53 (1.35)	140 (3.57)	193 (4.92)
No ART, no IPT	645.9	52 (8.05)	2 (0.31)	54 (8.21)
No ART, treated with IPT	77.0	4 (5.20)	0 (0)	4 (5.20)
On ART, treated with IPT	845.7	1 (0.12)	6 (0.71)	7 (0.83)
Total	5491.2	110 (2.00)	148 (2.70)	258 (4.70)

***ART*** Antiretroviral Therapy, ***IPT*** Isoniazid Preventive Therapy, ***n*** Number of events, ***PY*** Person Year, ***TB*** Tuberculosis

**Table 4:** The crude and adjusted estimate of cox regression model fitting IPT and ART treatment status as time dependent covariates for TB or death in HIV cohort of Dilla University hospital from January 2007 to August 2013

Covariates		Crude HR 95 % CI	Adjusted HR^a^ 95% CI
Combination therapy On ART, no IPT		1	1
No ART, no IPT		1.08 (0.79, 1.47)	1.36 (0.97, 1.91)
No ART, treated with IPT		1.22 (0.45, 3.28)	1.86 (0.68, 5.10)
On ART, treated with IPT		0.35 (0.16, 0.77)	0.40 (0.18, 0.87)
**Covariates:**		**Crude HR 95 % CI**	Adjusted HR^a^ 95% CI
Gender (male)		1.44 (1.12, 1.83)	1.56 (1.18, 2.06)
Age (years)^b^		1.01 (1.00, 1.03)	1.01 (0.99, 1.02)
Baseline body weight (Kg)^c^		0.98 (0.97, 0.99)	0.99 (0.97, 1.00)
Baseline CD4 count (Cells/mm^3^)^d^		1.00 (1.00, 1.01)	1.00 (0.999, 1.001)
Cotrimoxazole (ever use)		0.56 (0.43, 0.72)	0.72 (0.51, 1.02)
Baseline Function functional status	Ambulatory	1	1
	Bedridden	2.53 (1.72, 3.72)	2.47 (1.62, 3.75)
	Working	0.56 (0.43, 0.73)	0.67 (0.50, 0.89)
Baseline WHO HIV disease stage	Stage I	1	1
	Stage II	1.12 (0.54, 2.31)	1.11 (0.53, 2.30)
	Stage III	2.46 (1.33, 4.58)	2.14 (1.13, 4.06)
	Stage IV	3.71 (1.87, 7.37)	2.25 (1.07, 4.74)
Patients transferred from other hospital		0.33 (0.17, 0.64)	0.39 (0.18, 0.82)
Entry from	From the same facility	1	1
	Self-referred	0.61 (0.20, 1.85)	0.71 (0.23, 2.15)
	Referred from other facilities	0.61 (0.41, 0.87)	0.73 (0.48, 1.10)

^a^ **IPT & ART** were adjusted to the baseline covariates: gender, age, baseline body weight, baseline CD4 count, cotrimoxazole prophylaxis (ever use), baseline WHO functional status, baseline WHO stages, transfer history, and the entry point of care; ***HR*** Hazard Ratio, ***CI*** Confidence Interval, ***IPT*** Isoniazid Preventive Therapy, ***ART*** Antiretroviral Therapy; ^b^Age interval is in every five years; ^c^Baseline body weight is in every 10kgs; ^d^Baseline CD4 count is in every 50 cells/mm^3^

In the adjusted analysis, the same covariates appeared associated with risk of TB or death (Table 4). In comparison to ambulatory patients, bedridden patients had double the hazard to die or develop TB [aHR = 2.47; 95 % CI (1.62, 3.75)], and working patients had a 33 % lower hazard to die or develop TB [aHR = 0.67; 95 % CI (0.50, 0.89)]. With respect to the WHO staging, being in WHO stage II or higher was associated with an increased hazard of developing TB or death in comparison to patients in WHO stage I at baseline. Patients transferred to the University Hospital chronic care unit from primary health care units in the catchment area had a lower hazard to develop TB or die in comparison to patients who entered the cohort from different departments of the same hospital (aHR = 0.39; 95 % CI [0.18, 0.82]). The effects of age and baseline CD4 count on TB or death were more or less nominal (Table 4).

In the unadjusted time dependent Cox regression model, the combined treatment with IPT and ART lowered the hazard of TB or death by 65 % [HR = 0.35; 95 % CI (0.16, 0.77)] compared to ART alone and no such effect could be demonstrated for IPT in the absence of ART [HR = 1.22; 95 % CI (0.45, 3.28)]. The combined effect of IPT and ART lead to a 60 % reduction of the hazard of death or TB in comparison to ART without IPT after adjusting for baseline covariates [aHR = 0.40; 95 % CI (0.18, 0.87)]. It was not possible to reliably estimate the effect IPT in patients not treated with ART as there was little person time (77.0 PYs) available in this treatment category.

## Discussion

The estimated combined effect of IPT and ART on the risk of TB or death in HIV infected patients was a 60 % hazard reduction in comparison to ART without IPT after adjusting for baseline confounders. Due to the little patient time in the cohort for patients receiving IPT without concomitant ART, the isolated effect of IPT could not be reliably determined. Strikingly, only one fifth of the HIV patients included in this study actually received IPT.

These results are consistent with the results obtained in controlled clinical trials. In a recently updated Cochrane review of twelve clinical trials, IPT was associated with an overall TB risk reduction of 26 % in 8578 randomized HIV infected and TST positive participants [20]. Moreover, recent empirical evidence has shown that the combined benefit of ART and IPT was paramount in reducing the risk of TB and death (HR = 0.63, 95 % CI 0.41-0.94) [21, 31]. Further observational studies conducted in Brazil [32], South Africa [33], and Hong Kong [34] reported beneficial effect of combined therapy on TB. Although ART reduces HIV disease progression [35] and opportunistic infections including TB [36], the recent WHO policy guidelines do not precisely define how ART and IPT are best used together for optimal TB risk reduction [37]. The biological mechanism of combination therapy is that IPT reduces the burden of MTB [38, 39], whereas ART decreases the risk of developing active TB by improving immune function [40–42]. These mechanisms appear complementary and the combined use of these interventions might reasonably improve outcomes in HIV patients.

Of note, self-referred patients or those who were transferred from other health facilities had a lower risk of developing TB or dying. These patients started ART earlier compared to patients transferred to the HIV treatment unit from the same hospital. A plethora of studies reported that early diagnosis of HIV [43] and early therapy [44] play a significant role in HIV disease prevention and control. This study demonstrated that most events (TB or death) occurred in the first six months. This finding is consistent with a finding from Thailand where most of TB episodes observed within the first six months of enrolment [45].

The uptake of IPT in this study was lower than the global estimate (32.5 %), although there was no clear reason for this underutilization or under prescription [19]. Possible reasons may be the health care professionals’ skepticism about potential drug resistance [24] and subsequent underestimation of potential public health impact [46], as well as lack of adequate means to exclude a pre-existing TB infection prior to treatment initiation.

As opposed to most clinical trials, this study used the composite of TB or death as an outcome. Most trials found significant TB risk reduction and statistically non-significant effects in death prevention [21, 47]. As death and occurrence of TB are competing events, we chose to not separately assess the effect of IPT on these outcomes. Competing event analysis would be a means to reliability estimate the overall sub distribution hazard and event specific hazards [48]. The low number of TB or death cases in the IPT cohort, however, precluded a formal competing risks analysis. A strength of our study is the fit of a Cox regression model that includes time-varying exposures to accommodate changes in treatment status when IPT and/or ART are initiated. This possible source of so-called time dependent bias is often neglected, which can lead to overestimation of intervention effects [49–52].

Most limitations of this study follow from the retrospective study design, such as the inability of assessing time of HIV infection, precluding adjustment for length time bias [52]. All HIV patients were eligible to receive IPT as long as they fulfilled screening criteria [24, 25]. In this study, however, less than 20 % of the participants eligible for IPT received IPT, which may have created confounding by indication and this is difficult to address in observational studies [53]. The final model was adjusted for baseline covariates, although this does not control for effects of unmeasured confounders or time-varying confounders. Finally, many patients were lost to follow up and the underlying reasons for this could not be derived from the clinical charts or ART database. Importantly, there was little difference in the baseline characteristics between patients with and without loss to follow up and hence they were censored in the analysis.

## Conclusion

In this retrospective cohort study, the combination of IPT and ART reduced the hazard of TB or death in HIV-infected patients by 60 % in comparison to treatment with ART alone. Due to high levels of ART adoption, it was not possible to make strong conclusion about the benefits of IPT alone in the absence of ART. Contrary to the evidence arising from this and other studies, uptake of IPT in routine practice is very low (<20 %). We, therefore, recommend studies to determine the risk factors or predictors of the low IPT uptake, quantify the risk of isoniazid-resistant TB after IPT use, and elucidate the best strategies for (combined) initiation of IPT and ART.

## Abbreviations

AIDS: Acquired Immunodeficiency Syndrome
ART: Antiretroviral Therapy
CI: Confidence Interval
HIV: Human Immunodeficiency Virus
HR: Hazard Ratio
IPT: Isoniazid Preventive Therapy
SPSS: Statistical Package for Social Sciences
TB: Tuberculosis
TST: Tuberculin Skin Test
WHO: World Health Organization
